# Impact of FloTrac/EV1000-guided intraoperative hemodynamic optimization on postoperative outcomes in cardiac valve surgery: a randomized controlled trial

**DOI:** 10.1038/s41598-026-46157-x

**Published:** 2026-03-29

**Authors:** Sirirat Tribuddharat, Panaratana Ratanasuwan, Thepakorn Sathitkarnmanee, Netinai Chaimala, Lamphai Polsena, Patchareeporn Mantruad

**Affiliations:** 1 Department of Anesthesiology, Faculty of Medicine, Khon Kaen University, Khon Kaen, Thailand; 2 Nurse Anesthetist Division, Faculty of Medicine, Srinagarind Hospital, Khon Kaen University, Khon Kaen, Thailand; 3 Nurse Anesthetist Division, Queen Sirikit Heart Center of the Northeast, Faculty of Medicine, Khon Kaen University, Khon Kaen, Thailand

**Keywords:** Cardiac valve surgery, Hemodynamic optimization, FloTrac, EV1000, Postoperative outcomes, Goal-directed therapy, Cardiology, Diseases, Medical research

## Abstract

Cardiac valve surgery is associated with significant postoperative morbidity and mortality. This study evaluated the impact of intraoperative hemodynamic optimization using FloTrac/EV1000 on postoperative outcomes in patients undergoing cardiac valve surgery. In this single-center, prospective, randomized controlled trial, 82 patients undergoing elective cardiac valve surgery were randomly allocated to either FloTrac/EV1000 management (EV1000 group, *n* = 42) or conventional management (Control group, *n* = 40). The primary outcome was ICU length of stay. Secondary outcomes included mechanical ventilation duration, hospital length of stay, vasoactive drug requirements, fluid balance, and postoperative complications. The EV1000 group had significantly shorter ICU (30.2%) and hospital (13.6%) stay (*p* = 0.007 and 0.047, respectively) compared to the Control group. The EV1000 group required more vasoactive drugs during pre-bypass (*p* = 0.018) but fewer before ICU transfer (*p* = 0.003) and during their ICU stay (*p* < 0.05). The incidence of postoperative ventricular fibrillation (0 vs. 15.0%, *p* = 0.011), bradycardia (11.9 vs. 35.0%, *p* = 0.016), atrial fibrillation with rapid ventricular response (14.3 vs. 25.0%, *p* = 0.032), acute respiratory distress syndrome (0 vs. 5.0%, *p* = 0.045), and acute kidney injury (0 vs. 5.0%, *p* = 0.045) was lower in the EV1000 group. Goal-directed hemodynamic management using FloTrac/EV1000 monitoring in cardiac valve surgery was associated with shorter ICU and hospital length of stay, reduced postoperative vasoactive drug requirements, and fewer postoperative complications compared with conventional management. Whether this benefit derives from the monitoring technology, the structured hemodynamic algorithm, or their combination warrants confirmation in future multicenter trials.

Trial registration: NCT04292951 (The full date of first registration on https://ClinicalTrials.gov: March 1, 2020).

## Introduction

Cardiac valve surgery elicits a more pronounced systemic inflammatory response, as evidenced by higher interleukin-6 (IL-6) levels compared to coronary artery bypass grafting (CABG)^1^, and remains associated with considerable postoperative morbidity and mortality. The 30-day mortality is about 4–6%, nearly two-fold higher than CABG^2^, with complications occurring in up to 0.7–3.5% per patient-year^3^. Major postoperative complications include atrial fibrillation (30–64%)^4^, acute kidney injury (10–30%)^5^, respiratory complications (10–30%)^6^, and coagulopathy (5–15%)^7^. Specifically, post-valve surgery atrial fibrillation occurs in 30–50% of patients, potentially prolonging hospital stay and increasing healthcare costs^8^. Acute kidney injury after valve surgery is associated with a 5-fold increase in mortality risk^5^, while major bleeding complications necessitating reoperation occur in 2–8% of cases^9^.

Optimal perioperative hemodynamic management is crucial for improving outcomes; however, standardized protocols remain a topic of debate. The increasing complexity of valve procedures, often combined with CABG, poses additional challenges to intraoperative hemodynamic stability^10^.

Goal-directed therapy (GDT) using minimally invasive cardiac output monitoring has shown promise in reducing complications and length of stay in major surgery^11^. However, the unique physiological changes during cardiopulmonary bypass and the specific hemodynamic targets for different valve pathologies create uncertainty about optimal management strategies. Traditional monitoring approaches rely heavily on static parameters and clinical experience, which may not adequately reflect the dynamic nature of cardiac performance during and after valve surgery^12^.

Currently, two commonly utilized systems for minimally invasive cardiac output monitoring are the FloTrac/EV1000 and PiCCO. Both systems have demonstrated comparable performance in predicting fluid responsiveness^13^. The FloTrac/EV1000 system is preferred for its calibration-free operation, whereas the PiCCO system requires calibration via the thermodilution technique.

The FloTrac/EV1000 system (Edwards Lifesciences, Irvine, CA, USA) provides continuous cardiac output monitoring and dynamic parameters via arterial waveform analysis, offering potential advantages for guiding fluid and vasoactive (inotropes/vasopressors/vasodilators) therapy^14,15^. Stroke volume variation (SVV) was initially validated as a predictor of fluid responsiveness in closed-chest patients, prompting caution about its use in open-chest settings. However, more recent research has demonstrated that SVV, along with pulse pressure variation (PPV), remains a reliable tool for assessing fluid responsiveness even in patients undergoing open-chest or open-pericardial procedures^16,17^. Recent studies have demonstrated its efficacy in coronary artery bypass grafting (CABG), where its use was associated with reduced vasoactive drug requirements and shorter intensive care unit (ICU) stays^14^. In off-pump CABG procedures, hemodynamic optimization guided by FloTrac/EV1000 was associated with a shorter ICU stay and reduced hospital length of stay compared to conventional monitoring^15^. While these findings are promising for cardiac surgery in general, evidence specifically addressing its impact on valve surgery outcomes remains limited.

A recent meta-analysis suggests that protocol-driven hemodynamic optimization can reduce postoperative complications, particularly in high-risk cardiac surgical patients^18^. However, the optimal monitoring platform and specific intervention thresholds remain undefined. The FloTrac/EV1000 system’s ability to provide real-time SVV, stroke volume index (SVI), cardiac index (CI), and systemic vascular resistance index (SVRI) measurements without pulmonary artery catheterization may offer advantages for guiding perioperative management.

This randomized controlled trial evaluated the effects of FloTrac/EV1000-guided intraoperative hemodynamic optimization on postoperative outcomes in cardiac valve surgery. The primary outcome was ICU length of stay. Secondary outcomes included duration of mechanical ventilation, total hospital stay, vasoactive drug requirements, and postoperative complications. We hypothesized that a GDT approach, targeting SVV, CI, and SVRI, would reduce vasoactive drug requirements, shorten ICU length of stay, and decrease postoperative complications compared to conventional management.

## Methods

### Study design and patient population

This prospective randomized controlled trial was approved by the Khon Kaen University Ethics Committee in Human Research (IRB: HE611321, September 11, 2019) and registered at ClinicalTrials.gov (NCT04292951, March 1, 2020) before commencement. The study adhered to the Declaration of Helsinki, ICH GCP guidelines, and CONSORT reporting standards. Written informed consent was obtained from all participants.

The trial included two groups of participants randomized in a 1:1 ratio to either the FloTrac/EV1000-guided hemodynamic management (EV1000 group) or the conventional management (Control group). Randomization was performed using a computer-generated block-of-four sequence, with allocation concealment ensured via sealed opaque envelopes. Inclusion criteria were: age 18–80 years, elective cardiac valve surgery (with or without concomitant CABG) at Srinagarind Hospital or Queen Sirikit Heart Center of the Northeast, Khon Kaen University, Khon Kaen, Thailand, and American Society of Anesthesiologists (ASA) classification 2–4. Patients were excluded if they required emergency surgery (insufficient time for informed consent, baseline hemodynamic instability), redo surgery (prolonged operative time, increased bleeding risk, hemodynamic instability, and potential protocol non-adherence), preoperative intra-aortic balloon pump support (distorted arterial waveforms precluding accurate FloTrac/EV1000 measurements), or had severe pulmonary hypertension (right ventricular dysfunction and pulmonary vascular disease impairing the accuracy of arterial waveform–derived cardiac output). Blinding of patients and postoperative outcome assessors was implemented.

### Anesthesia and monitoring

All patients received standardized anesthetic care in accordance with institutional protocols. Standard monitoring included electrocardiography, pulse oximetry, non-invasive blood pressure measurement, invasive blood pressure (IBP), central venous pressure (CVP), capnography, nasopharyngeal temperature monitoring, and urine output assessment. Radial artery cannulation was performed in all patients. In the Control group, the arterial line was connected to a standard pressure transducer for IBP monitoring. In the EV1000 group, a FloTrac transducer connected to the EV1000 monitor (Edwards Lifesciences, Irvine, CA, USA) was used to measure IBP, SVV, SVI, and CI. To ensure comparability, all measured values were normalized to the patient’s body surface area using index values in this study. Internal jugular/subclavian vein cannulation was also performed in all patients. In the Control group, the catheter was connected to a standard pressure transducer for CVP measurement. In contrast, the EV1000 group utilized a pressure transducer integrated with the FloTrac/EV1000 system to monitor SVRI. During cardiopulmonary bypass (CPB), the absence of a pulsatile arterial waveform precludes FloTrac monitoring.

Anesthesia was induced with titrated propofol (1.5-2 mg/kg) or etomidate (0.2–0.3 mg/kg), and fentanyl (2–5 µg/kg) intravenously, followed by endotracheal intubation facilitated with cisatracurium (0.2 mg/kg) intravenously. Mechanical ventilation was set with a tidal volume of 10 mL/kg ideal body weight and a respiratory rate of 12 breaths/min, adjusted to maintain an end-tidal CO₂ concentration of 30–35 mmHg. Anesthesia was maintained with 50–60% oxygen in air and 1–2% sevoflurane or 3–6% desflurane, titrated to 0.7–0.8 minimum alveolar concentration (MAC). CPB was initiated after heparinization (3–4 mg/kg via the central venous catheter) with an activated clotting time (ACT) > 480 s, maintained above 400–480 s with supplemental heparin (0.5-1 mg/kg). Moderate hypothermia (32 °C) was maintained during CPB. Cardioplegia was administered via an aortic root catheter, with supplemental doses as needed at the surgeon’s discretion. Mean arterial pressure (MAP) was maintained between 50 and 75 mmHg during CPB. Protamine (0.7-1 mg per 1 mg of pre-CPB heparin) was slowly administered intravenously for heparin reversal after CPB weaning. Postoperatively, all patients were transferred to the ICU, intubated, and supported with a transport ventilator, after which standard ICU care was provided. In the ICU, patients were either maintained on mechanical ventilation or extubated to spontaneous breathing based on established extubation criteria. Extubation criteria included: adequate consciousness and motor strength, cardiovascular stability, a PaO_2_/FiO_2_ ratio ≥ 250 mmHg, and a respiratory rate of 10–25 breaths/min. ICU discharge criteria were: adequate consciousness and neurological status, cardiovascular stability without inotropic or vasopressor support, stable respiratory status with < 60% oxygen requirement, and no need for ICU monitoring.

ICU discharge decisions were made by attending ICU physicians who were blinded to intraoperative group allocation, and all discharged patients met the above criteria. Residual clinician discretion in the precise timing of discharge cannot be entirely excluded and represents a potential source of detection bias.

Hospital discharge criteria included: cardiovascular and respiratory stability, removal of all drains and catheters, normal ambulation, absence of infection or serious complications, wound suture removal, and tolerance to a normal diet.

### Intraoperative hemodynamic management protocol

**The control group**: Hemodynamic management was at the discretion of the attending anesthesiologists, who administered fluids, inotropes, and/or vasoactive medications as needed to maintain the following targets: MAP 65–90 mmHg, CVP 8–12 mmHg, urine output ≥ 0.5 mL/kg/h, SpO₂ ≥ 95%, and hematocrit 26–30% (22–25% during CPB), according to standard institutional practices, without rigid protocolized algorithm. Hourly arterial blood gas analysis and electrolyte monitoring with appropriate correction were performed.

**Fig. 1:**
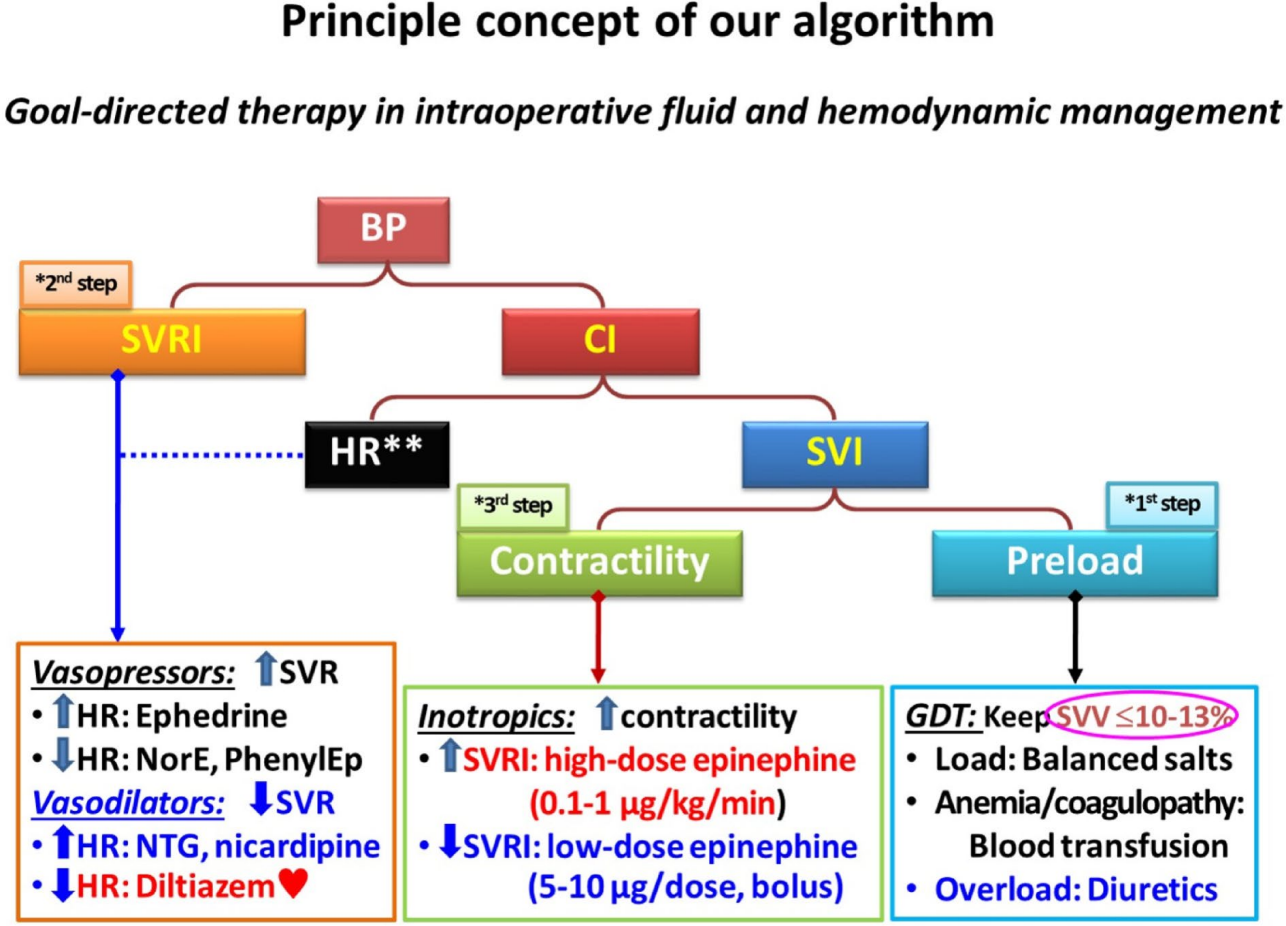
Algorithm for goal-directed therapy (GDT) in intraoperative fluid and hemodynamic management. *BP*, blood pressure; *CI*, cardiac index; *SVRI*, systemic vascular resistance index; *SVI*, stroke volume index; *HR*, heart rate; *SVV*, stroke volume variation; *NorE*, norepinephrine; *PhenylEp*, phenylephrine; *NTG*, nitroglycerine; *MAP*, mean arterial pressure.

**The EV1000 group**: Hemodynamic management aimed to achieve the same targets (MAP 65–90 mmHg, urine output ≥ 0.5 mL/kg/h, SpO₂ ≥ 95%, and hematocrit 26–30% [22–25% during CPB]). When these targets were not met, management was guided by a structured algorithm using FloTrac/EV1000-derived parameters (Fig. 1).

**To summarize**: FloTrac/EV1000-derived parameters (SVV, CI, SVRI) were assessed and acted upon only when conventional hemodynamic targets (MAP and urine output) were not achieved. Within the algorithm, SVV guided initial preload optimization; SVRI guided afterload assessment only if hypotension persisted after preload correction; and CI guided contractility assessment only if hypotension remained after both preload and afterload management. Advanced parameters were not independently corrected when conventional targets were within acceptable ranges.

#### Step 1: preload assessment

Preload adequacy was initially assessed using SVV, with a threshold of 10–13%. If SVV > 10–13%, a fluid challenge with 50–100 mL of crystalloid over 5–10 min was administered. In patients with anemia or coagulopathy, blood or blood components were transfused as needed. For those with signs of volume overload, diuretic therapy was considered.

Following preload optimization (SVV ≤ 10–13%), patients were classified based on MAP. If MAP ≥ 65 mmHg, the patient was considered purely hypovolemic. If MAP remained below 65 mmHg, further management was guided by assessments of afterload and contractility to determine the appropriate intervention.

#### Step 2: afterload assessment

For low SVRI (< 1,800 dynes/sec/cm^5^/m^2^):

- If systolic arterial pressure (SAP) < 90 mmHg or MAP < 65 mmHg with heart rate (HR) < 70 bpm, administer an intravenous ephedrine bolus of 3–6 mg, which will stimulate both α and β receptors, increasing contractility, HR, and afterload.
- If SAP < 90 mmHg or MAP < 65 mmHg with HR > 70 bpm, administer norepinephrine (4–8 µg) or phenylephrine (50–100 µg) as an intravenous bolus, which will affect mainly the α receptor, resulting in an increase in afterload, followed by a reflex decrease in HR.

For high SVRI (> 2,500 dynes/sec/cm^5^/m^2^):

- If SAP > 140 mmHg or MAP > 90 mmHg with HR < 70 bpm, administer an intravenous nicardipine bolus (0.5-2 mg) or initiate a nitroglycerin infusion, which are direct vasodilators, resulting in a decrease in afterload, followed by a reflex increase in HR.
- If SAP > 140 mmHg or MAP > 90 mmHg with HR > 70 bpm, administer an intravenous diltiazem bolus of 2–5 mg, which will decrease both afterload and HR.

#### Step 3: contractility assessment

For low CI (< 2.0–2.2 L/min/m^2^):

- If SVRI < 1,500 dynes/sec/cm⁵/m² with SAP < 90 mmHg or MAP < 65 mmHg, administer an intravenous high-dose epinephrine infusion (> 0.2 µg/kg/min), which will stimulate both α and β receptors, increasing contractility and afterload.
- If SVRI > 2,500 dynes/sec/cm⁵/m² with SAP < 90 mmHg or MAP < 65 mmHg, administer an intravenous low-dose epinephrine bolus (5–10 µg). At this low dose, epinephrine predominantly stimulates β₁- and β₂-adrenergic receptors, increasing stroke volume and cardiac output while reducing afterload.

For high CI (> 5.5–6.0 L/min/m²) indicating a hyperdynamic state in septic shock:

- If SVRI < 1,200 dynes/sec/cm⁵/m² with SAP < 90 mmHg or MAP < 65 mmHg, initiate an intravenous infusion of norepinephrine or phenylephrine, along with treatment for the underlying cause of septic shock.

### Postoperative management

Following surgery, the EV100 monitor was removed. The FloTrac was replaced by a standard invasive blood pressure transducer. Patients were transferred to the ICU for uniform standard care, and ICU staff were unaware of the intraoperative intervention.

### Data collection and outcomes

The primary outcome was ICU length of stay. Secondary outcomes included mechanical ventilation duration, total hospital length of stay, and vasoactive drug requirements (types and numbers of agents) across predefined perioperative phases—pre-CPB, post-CPB, at ICU transfer, and during the ICU stay. Additional secondary outcomes included fluid balance and postoperative complications. Arrhythmic complications comprised ventricular fibrillation (VF), bradycardia (heart rate < 60 bpm), atrial fibrillation (AF) with rapid or slow ventricular response (ventricular rate > 100 bpm or < 60 bpm), and supraventricular tachycardia (SVT; narrow-complex tachycardia > 100 bpm). Non-arrhythmic complications included acute respiratory distress syndrome (ARDS; PaO₂/FiO₂ ≤ 300 mmHg), acute kidney injury (AKI; increase in serum creatinine ≥ 0.3 mg/dL within 48 h or ≥ 1.5 times baseline), complete heart block, reintubation, coagulopathy, requirement for temporary pacemaker insertion, and thrombocytopenia.

### Statistical analysis

The sample size was calculated to detect a 25% reduction in ICU length of stay after cardiac surgery, based on data from a previous, most relevant study reporting a mean ICU stay of 4.9 ± 1.8 days^12^. Assuming a significance level (α) of 0.05, a power of 80%, and accounting for a 20% dropout rate, 40 patients per group were required.

The normality of continuous data was assessed using the Shapiro-Wilk test. Normally distributed data are presented as mean ± standard deviation (SD) and were compared using the unpaired Student’s t-test. Non-normally distributed data are presented as median (interquartile range) and were compared using the Mann-Whitney U test. Categorical data are presented as number (%) and analyzed using the appropriate chi-squared (χ²) test or Fisher’s exact test. The primary outcome is reported as the mean difference with a 95% confidence interval (CI). Statistical significance was defined as *P* < 0.05. All analyses were conducted using SPSS 16.0 (SPSS Inc., Chicago, IL, USA).

## Results

**Fig. 2:**
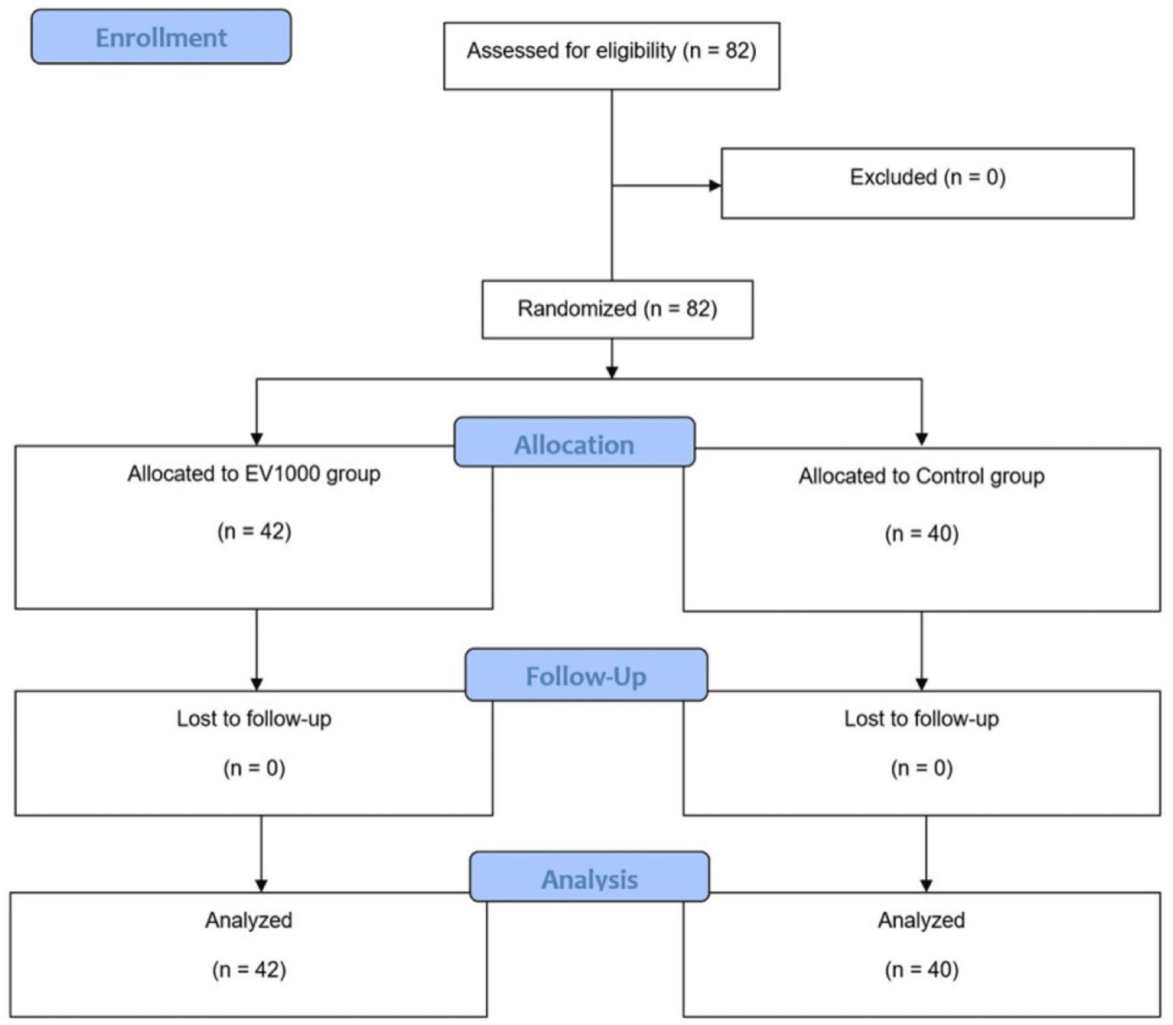
CONSORT diagram of the study.

A total of 82 patients were recruited between March 2021 and February 2022, with 42 patients in the EV1000 group and 40 in the Control group (Fig. 2). Baseline characteristics and preoperative laboratory values were generally comparable between the groups (Table 1).

**Table 1:** Patient characteristics and perioperative clinical data (n = 82).

	EV1000 (*n* = 42)	Control (*n* = 40)	*p* value
Sex: male/female	28 (66.7)/14 (33.3)	26 (65.0)/14 (35.0)	0.872
Age (y)	61.2 ± 10.5	63.0 ± 8.6	0.397
Body weight (kg)	60.5 ± 9.1	59.7 ± 9.7	0.701
Height (cm)	161.6 ± 8.9	163.5 ± 9.0	0.339
Ejection fraction (%)	58.6 ± 15.3	61.4 ± 15.4	0.411
Type of operation			0.442
MV repair/MVR	5 (11.9)/6 (14.3)	2 (5.0)/1 (2.5)	
MV repair+CABG	7 (16.7)	6 (15.0)	
MVR & TVA/MVR & TVA+CABG	4 (9.5)/2 (4.8)	4 (10.0)/1 (2.5)	
AVR	7 (16.7)	6 (15.0)	
AVR+CABG	10 (23.8)	5 (12.5)	
AVR & MV repair+CABG	1 (2.4)	1 (2.5)	
AVR & TVA/AVR & TVA+CABG	1(2.4)/1 (2.4)	0 (0)/1 (2.5)	
AVR & ascending aortic graft	1 (2.4)	0 (0)	
DVR & TVA ± LAA closure	1 (2.4)	1 (2.5)	
DVR/DVR+CABG	1 (2.4)/2 (4.8)	1 (2.5)/2 (5.0)	
TVA & maze procedure	0 (0)	1 (2.5)	
TVA & ASD closure	1 (2.4)	2 (5.0)	
TVA & ASD closure+CABG	0 (0)	1 (2.5)	
Functional class			0.846
2	24 (57.1)	25 (62.5)	
3	17 (40.5)	15 (37.5)	
4	1 (2.4)	0 (0)	
ASA classification			0.703
2	12 (28.6)	13 (32.5)	
3	30 (71.4)	27 (67.5)	
Underlying diseases			
Hypertension	20 (47.6)	18 (45.0)	0.812
Diabetes mellitus	14 (33.3)	11 (27.5)	0.567
Myocardial ischemia	24 (57.1)	19 (47.5)	0.382
Dyslipidemia	5 (11.9)	4 (10.0)	0.784
Atrial fibrillation	8 (19.0)	10 (25.0)	0.513
Chronic kidney disease	12 (28.6)	6 (15.0)	0.138
Old cardiovascular accident	2 (4.8)	3 (7.5)	0.596
Coagulopathy	6 (14.3)	6 (15.0)	0.927
Preoperative			
Creatinine (mg/dL)	1.6 ± 2.0	1.0 ± 0.3	0.058
Sodium (mEq/L)	138.9 ± 3.0	139.6 ± 2.4	0.245
Potassium (mEq/L)	4.2 ± 0.4	4.1 ± 0.4	0.261
Blood sugar (mg/dL)	122.1 ± 50.2	114.8 ± 42.3	0.478
Hemoglobin (g/dL)	12.4 ± 2.0	12.6 ± 1.9	0.644
Albumin (mg/dL)	4.2 ± 0.6	4.1 ± 0.4	0.380
Platelet (x10^9^/L)	232.7 ± 88.7	239.3 ± 86.6	0.734
INR	1.1 ± 0.3	1.0 ± 0.4	0.205
Lactate (mmol/L)	0.9 ± 0.3	1.0 ± 0.4	0.203
Anesthesia time (min)	380.5 ± 88.5	362.4 ± 101.6	0.386
CPB time (min)	145.5 ± 42.0	139.5 ± 50.2	0.552
Aortic cross-clamp (min)	98.3 ± 27.3	90.5 ± 35.1	0.258
Operation time (min)	318.5 ± 83.2	310.4 ± 98.4	0.683
Crystalloid intake (mL)	1,576.7 ± 600.5	1,538.2 ± 663.6	0.784
Blood loss (mL)	1,023.3 ± 248.8	1,056.6 ± 293.9	0.574
Urine output (mL)	1,091.3 ± 727.6	993.4 ± 612.7	0.513

Data are presented as mean ± SD or n (%).

*MV*, mitral valve; *MVR*, mitral valve replacement; *CABG*, coronary artery bypass graft; *TVA*, tricuspid valve annuloplasty; *AVR*, aortic valve replacement; *DVR*, double valve replacement; *LAA*, left atrial appendage; *ASD*, atrial septal defect; *INR*, international normalized ratio; *CPB*, cardiopulmonary bypass.

The distribution of surgical procedures was similar between the groups. Duration of anesthesia, CPB time, and aortic cross-clamp time were also comparable. The crystalloid intake, blood loss, and urine output were also similar between the groups (Table 1).

ICU length of stay, ventilator time, and hospital stay exhibited non-normal distributions, as confirmed by the Shapiro-Wilk test, and were compared using the Mann-Whitney U test. These outcomes are presented as both median (interquartile range) (for descriptive accuracy) and mean ± SD (to facilitate meta-analytic comparisons with existing literature that predominantly reports mean ± SD).

The EV1000 group had a significantly shorter ICU (30.2%) and hospital (13.6%) stay (*p* = 0.007 and 0.047, respectively). A post hoc power analysis indicated a statistical power of 85.6% for the primary outcome. Ventilation time was comparable between groups (Table 2).

**Table 2:** Postoperative outcomes (n = 82).

	EV1000 *n* = 42	Control *n* = 40	Mean difference	95% CI	*p* value
ICU stay (h)	42.0 (40.0–48.0)	49.0 (41.8–77.0)			0.007*
	44.6 ± 6.3	63.9 ± 39.9	-19.3	-31.8 to -6.8	0.002*
Ventilator time (h)	15.0 (12.0-16.3)	14.5 (2.3–18.0)			0.882
	13.3 ± 7.1	13.5 ± 12.1	-0.2	-4.5 to 4.1	0.924
Hospital stay (d)	11.0 (9.0-12.3)	11.5 (11.0-14.5)			0.047*
	11.4 ± 2.9	13.2 ± 4.0	-1.8	-3.3 to -0.3	0.021*

Data are presented as mean ± SD or median (interquartile range).

* *p* < 0.05, compared using Mann-Whitney U test or unpaired Student’s t-test.

*ICU*, intensive care unit.

**Fig. 3:**
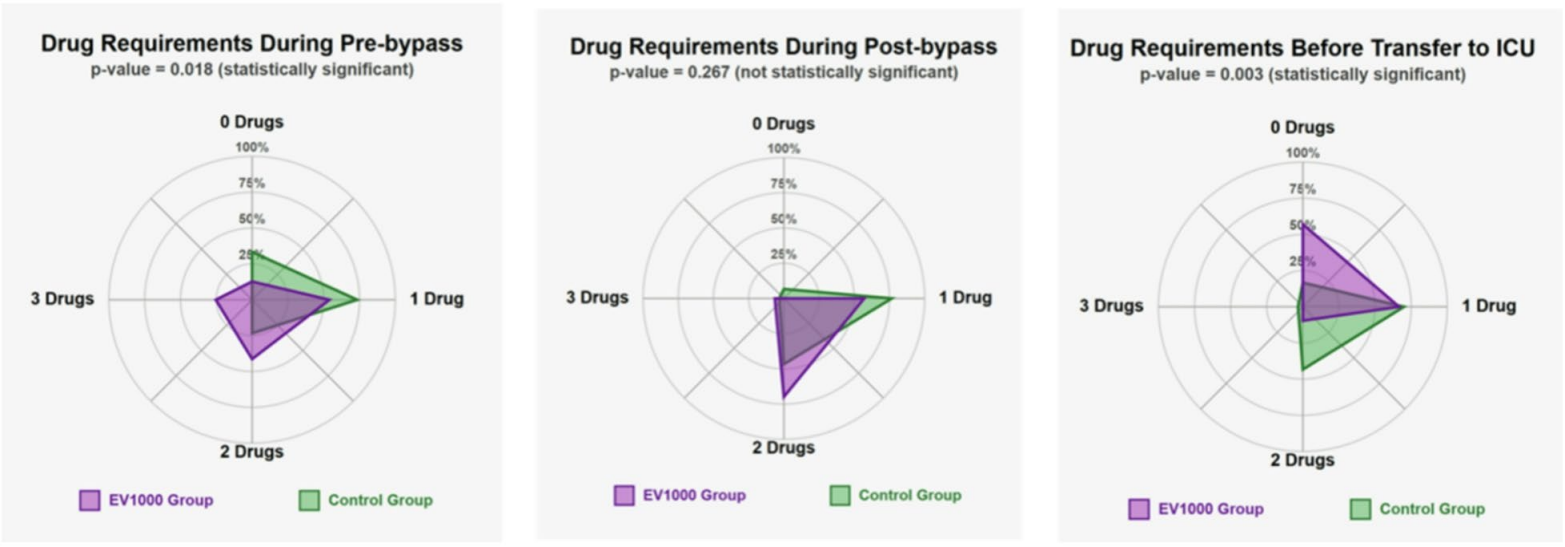
Drug requirements at different intraoperative stages in the operating room. The concentric circles represent the percent of patients, while each radial spoke indicates the number of drugs required (0–3 drugs). Values plotted on the graph show the distribution of patients according to their drug requirements.

**Fig. 4:**
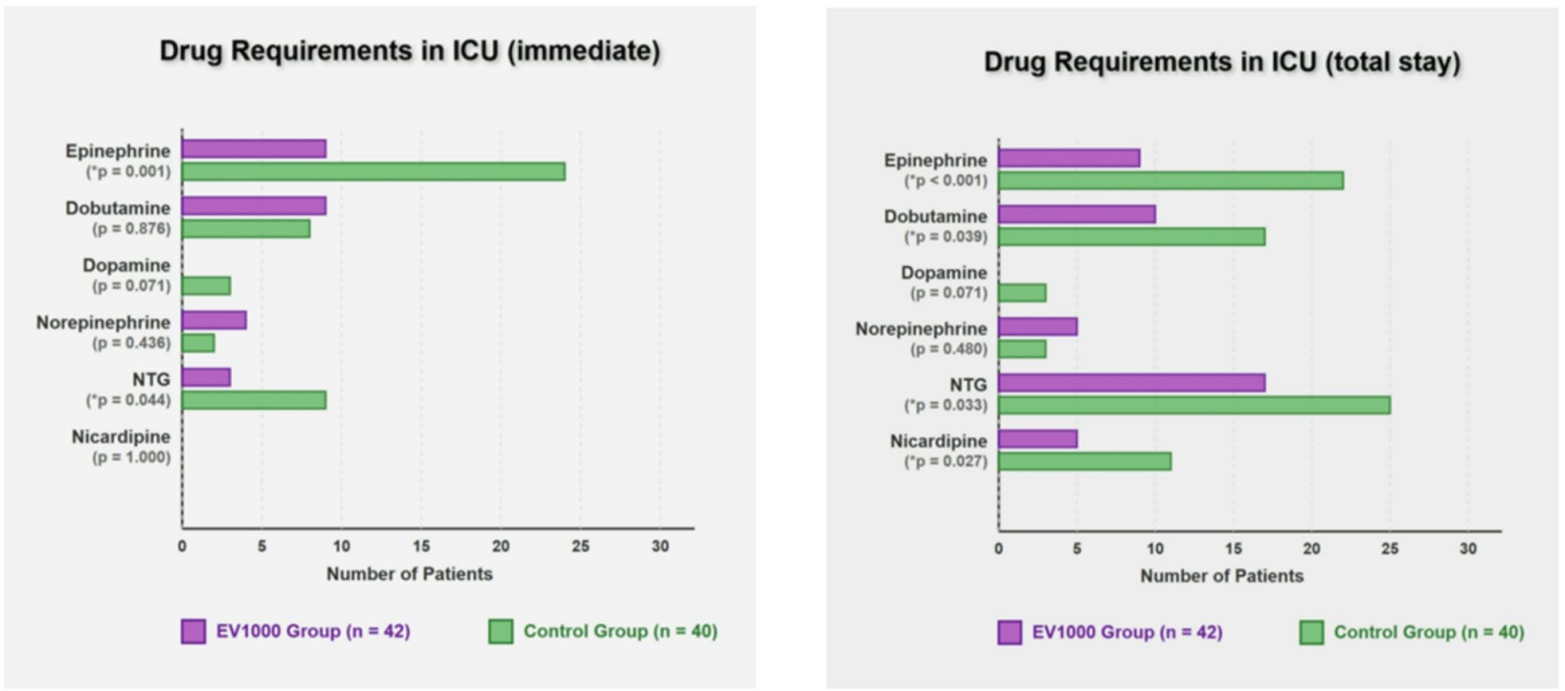
Drug requirements in the ICU during the immediate and total ICU stay. *NTG*, nitroglycerine.

Regarding vasoactive drug requirements in the operating room, the EV1000 group required more concurrent drugs during the pre-bypass phase, showed no significant difference post-bypass, and required fewer drugs before ICU transfer (Fig. 3). Immediately upon ICU admission, the EV1000 group required significantly less epinephrine and nitroglycerin (NTG). Over the total ICU stay, the EV1000 group required significantly less epinephrine, dobutamine, NTG, and nicardipine (Fig. 4).

**Fig. 5:**
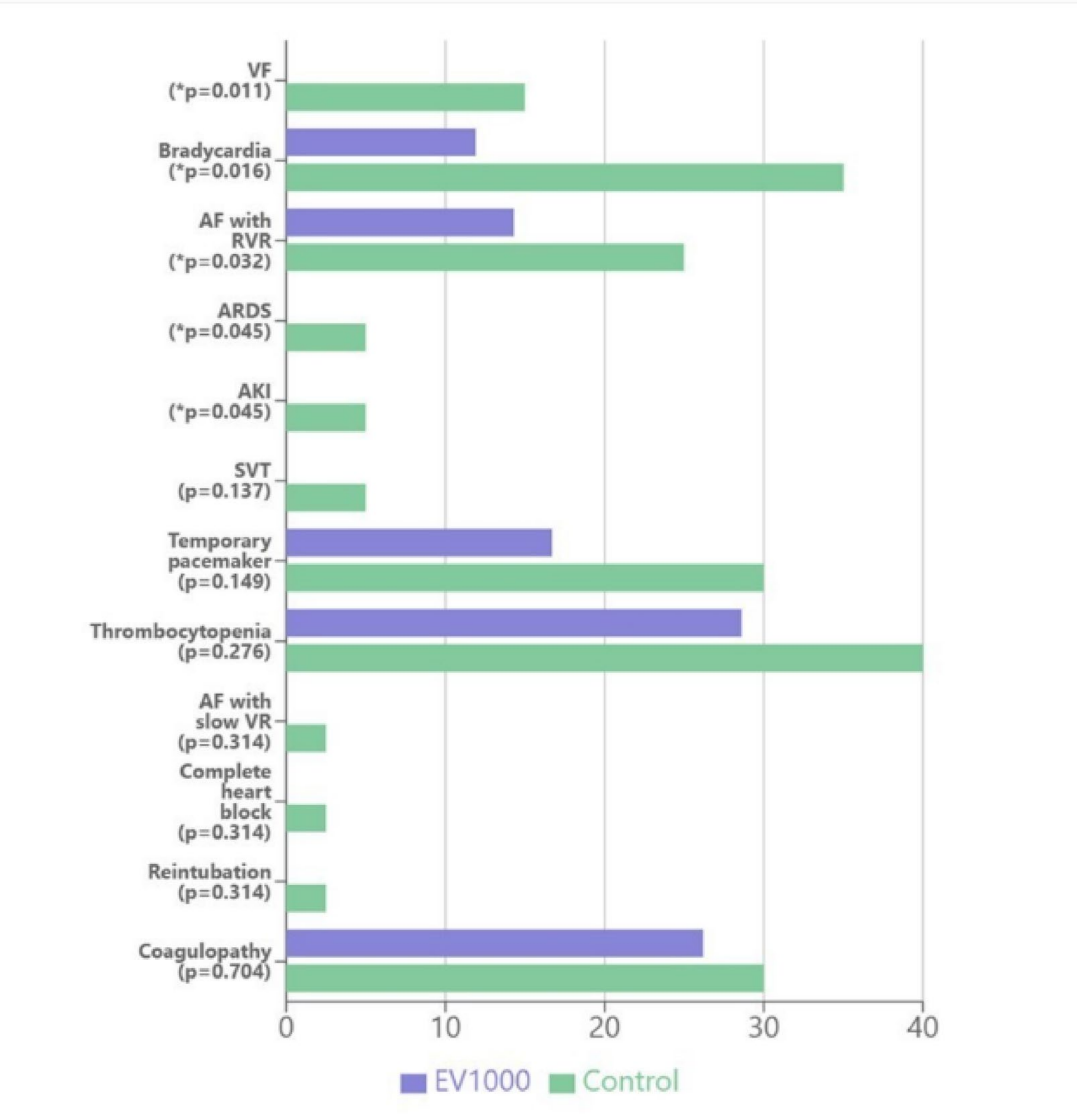
Postoperative complications. *VF*, ventricular fibrillation; *AF with RVR*, atrial fibrillation with rapid ventricular response; ARDS, acute respiratory distress syndrome; AKI, acute kidney injury; SVT, supraventricular tachycardia; AF with slow VR, atrial fibrillation with slow ventricular response.

The EV1000 group demonstrated a lower incidence of VF, bradycardia, AF with rapid ventricular response (RVR), ARDS, and AKI. Other complications, including SVT, temporary pacemaker use, thrombocytopenia, AF with slow ventricular response, complete heart block, reintubation, and coagulopathy, showed no significant differences between groups (Fig. 5).

## Discussion

This randomized controlled trial demonstrated that GDT using FloTrac/EV1000-guided hemodynamic management in cardiac valve surgery resulted in shorter ICU and hospital stays, reduced vasoactive drug requirements, and fewer postoperative complications compared to conventional management. The 19.3-hour reduction in ICU stay and 1.8-day decrease in hospital stay represent clinically significant findings. Although the sample size of this study was derived from a small, single-center randomized study without a formal a priori power calculation, a post hoc power analysis indicated a statistical power of 85.6%, suggesting the sample size was adequate for the primary outcome.

The pattern of vasoactive drug use varied notably between groups. While the EV1000 group required more vasoactive support during the pre-bypass period, they needed significantly fewer drugs before ICU transfer and throughout their ICU stay. This suggests that early hemodynamic optimization during the pre-bypass period may enhance cardiovascular stability in the postoperative period. Reduced vasoactive drug requirements in the ICU indicate better hemodynamic stability, likely contributing to shorter ICU and hospital stays.

A mechanistic pathway linking intraoperative hemodynamic optimization to shorter ICU stay is supported by the pattern of secondary outcomes. The EV1000 group had significantly lower rates of postoperative ventricular fibrillation (0% vs. 15.0%, *p* = 0.011), bradycardia (11.9% vs. 35.0%, *p* = 0.016), AF with rapid ventricular response (14.3% vs. 25.0%, *p* = 0.032), ARDS (0% vs. 5.0%, *p* = 0.045), and AKI (0% vs. 5.0%, *p* = 0.045). Each of these complications is a recognized driver of prolonged ICU stay in cardiac surgical patients: arrhythmias such as VF and AF with RVR require hemodynamic monitoring, antiarrhythmic therapy, and potentially cardioversion, all of which sustain the need for ICU-level care, while ARDS and AKI mandate ventilatory support and renal monitoring, respectively, directly delaying discharge. Furthermore, the EV1000 group required significantly fewer vasoactive drugs before ICU transfer (*p* = 0.003) and throughout the ICU stay (*p* < 0.05). Our pre-specified ICU discharge criteria required cardiovascular stability without inotropic or vasopressor support; therefore, reduced vasoactive requirements in the EV1000 group represent a criterion-linked mechanistic link between intraoperative hemodynamic optimization and shorter ICU LOS. Nevertheless, several alternative explanations should be considered: (1) the benefit may partly reflect protocolized versus non-protocolized care rather than the monitoring technology per se; (2) greater postoperative vasoactive use in the Control group may partly reflect inter-physician practice variation; (3) unmeasured confounders cannot be excluded in a single-center trial of this size; and (4) the absence of a statistically significant difference in mortality between groups limits the strength of causal inference. These considerations underscore the need for cautious interpretation of the observed difference in ICU LOS. The later discharge of the Control group from the ICU is mechanistically consistent with the higher incidence of postoperative arrhythmias, ARDS, AKI, and the greater ongoing vasoactive drug requirements observed in that group—each of which directly delayed fulfilment of the pre-specified ICU discharge criteria, particularly the requirement for cardiovascular stability without inotropic or vasopressor support.

Regarding postoperative complications, VF is an uncommon postoperative complication, with a reported incidence of approximately 2.7% following CABG. The underlying mechanisms of VF remain incompletely understood. Elevated catecholamine levels and autonomic imbalance during the early postoperative recovery period may contribute to the initiation of dysrhythmias^19^. However, the incidence following cardiac valve surgery is expected to be higher due to greater ventricular manipulation, longer CPB duration, pre-existing structural heart disease (including ventricular hypertrophy, fibrosis, and chamber dilatation), and the more pronounced systemic inflammatory response associated with valve surgery, all of which promote electrical heterogeneity and re-entrant arrhythmogenesis^1^. The 15.0% incidence in the Control group of the present study is therefore mechanistically plausible in this context. In addition, the significantly greater postoperative epinephrine use in the Control group (*p* < 0.001) may have further predisposed to VF through heightened beta-adrenergic stimulation and increased ventricular automaticity, consistent with established arrhythmogenesis mechanisms in the postoperative cardiac surgical population. Postoperative bradycardia occurred in 5.25% of patients, with half requiring temporary cardiac pacing. Risk factors for postoperative bradycardia include the adequacy of intraoperative myocardial perfusion^20^. The lower incidence of postoperative arrhythmias (VF, bradycardia, and AF with RVR) in the EV1000 group is particularly noteworthy. The reduced incidence of postoperative AF with RVR (14.3% vs. 25.0%) was consistent with the findings of Tribuddharat et al., who showed that GDT using FloTrac/EV1000 reduced the incidence of AF with RVR in patients undergoing CABG and off-pump coronary artery bypass (OPCAB)^14,15^. This reduction may be attributed to improved perioperative hemodynamic optimization, as unstable hemodynamics and suboptimal tissue perfusion are well-recognized risk factors for postoperative arrhythmias^21^. The greater postoperative use of epinephrine in the Control group (*p* < 0.001), may have increased beta-adrenergic stimulation, potentially contributing to the higher incidence of VF (15% vs. 0%) and AF with RVR (25% vs. 14.3%) observed. This difference, possibly due to discretionary management practices, underscores the need for standardized postoperative protocols.

The incidence of ARDS in the perioperative period of cardiac surgery has been reported to range from 0.4% to 8.1%^22^. The complete absence of ARDS in the EV1000 group in this study highlights the potential benefit of hemodynamic optimization using FloTrac/EV1000.

Our study showed a greater reduction in AKI (0% vs. 5%) than the previous report by Meersch et al. (55.1% vs. 71.7%)^23^. This improved renal outcome might be attributed to our strict adherence to hemodynamic optimization protocols in the EV1000 group. This finding supports the idea that optimized hemodynamic management can help preserve organ function, particularly during the vulnerable perioperative period^24^.

Since the FloTrac/EV1000 system derives hemodynamic information from the arterial waveform, it is limited during the CPB period, as the absence of an arterial waveform prevents the system from displaying any data.

An important consideration in interpreting the present findings is that the EV1000 group followed a structured, stepwise hemodynamic algorithm, whereas the Control group was managed without a predefined protocol. This study, therefore, compares protocolized FloTrac/EV1000-guided care with non-protocolized conventional management, and, given this design, it is not possible to determine whether the observed benefits derive from the advanced monitoring technology itself, the structured decision-making algorithm, or both. Future studies should incorporate a protocolized conventional management arm to isolate these effects.

Our findings have important clinical implications for perioperative management in cardiac valve surgery. They align with several recent studies on minimally invasive cardiac output monitoring in cardiac surgery. GDT using FloTrac/EV1000 reduced ICU stay and the use of vasopressor or inotropic drugs in patients undergoing cardiac surgery, including CABG and OPCAB surgery^12,14,15,25,26^. The 19.3-hour reduction in ICU stay observed in our study was comparable to the findings of Kapoor et al., who reported a mean reduction of 2.3 days in moderate to high-risk cardiac surgery patients^12^. In patients undergoing CABG and OPCAB, Kapoor et al. reported reductions in ICU stay of 0.33 and 1.7 days, respectively^25,26^, while Tribuddharat et al. reported reductions of 29.5 h and 1.3 days, respectively^14,15^.

Our structured GDT algorithm provided a systematic approach to hemodynamic management by stepwise assessing preload, contractility, and afterload (Fig. 1). By incorporating specific intervention thresholds, including HR and SVRI for drug selection, the algorithm enabled precise hemodynamic optimization. This approach differs from a previous expert review that relied on more generalized hemodynamic targets, such as pulse pressure variation, pleth variability index, and changes in stroke volume during a mini-fluid challenge or passive leg raising^27^. The observed pattern of vasoactive drug use—with more intensive optimization pre-bypass followed by reduced postoperative requirements—reflects the structured nature of the algorithm. The higher initial vasoactive requirements in the EV1000 group likely resulted from strict adherence to specific hemodynamic thresholds, including MAP > 65 mmHg, CI > 2.2 L/min/m², and SVV < 10–13%. We prefer to give vasoactive drugs as titrated bolus doses when indicated to restore the deviated parameters to normal ranges as soon as possible. Our principle of “***identify the right causes***, ***give the right drugs***, ***at the right time***” ensures optimal organ perfusion throughout the perioperative period, thereby minimizing postoperative vasoactive drug requirements and reducing complications. This proactive approach to hemodynamic management may explain the observed improvements in postoperative stability and the reduction in complications and ICU stay.

The investment in advanced hemodynamic monitoring technology appears justified by reductions in complications and length of stay, which likely lead to lower healthcare costs and improved patient outcomes^28^. The implementation of FloTrac/EV1000-guided management protocols could be particularly beneficial for high-risk cardiac surgical patients, where precise hemodynamic optimization is crucial.

### Limitations

The main limitations of this study are its single-center design and relatively small sample size. Additionally, the inability to blind the anesthesiologists to group allocation may have introduced some bias. While the observed reduction in ICU stay (30.2%) exceeded our 25% estimate, the optimistic effect size assumption may have led to insufficient power for secondary outcomes. Future multicenter trials with conservative effect sizes and larger sample sizes would strengthen the evidence. Given the demonstrated intraoperative benefits of the FloTrac/EV1000 system, its application in postoperative ICU monitoring warrants consideration. However, advanced systems such as PiCCO, which provide measurements of extravascular lung water and global end-diastolic volume, may offer complementary data to optimize fluid management and address respiratory complications in the ICU. Future studies are needed to compare the performance and clinical utility of these monitoring systems in the postoperative setting.

Despite the application of standardized ICU discharge criteria, residual clinician discretion in the precise timing of discharge cannot be entirely excluded, representing a potential source of detection bias in the primary outcome measurement.

A further limitation is that the benefit observed in the EV1000 group cannot be attributed solely to the FloTrac/EV1000 monitoring platform itself. The concurrent use of a predefined stepwise hemodynamic algorithm represents a co-interventional element that may independently contribute to the improved outcomes. The relative contributions of the monitoring technology and the structured protocol cannot be disentangled in the present study design, which represents an important direction for future research.

## Conclusions

Goal-directed hemodynamic management using FloTrac/EV1000 monitoring in cardiac valve surgery was associated with shorter ICU and hospital length of stay, reduced postoperative vasoactive drug requirements, and fewer postoperative complications compared to conventional management. The structured algorithm incorporating SVV, CI, SVRI, and HR parameters provided an effective framework for optimizing hemodynamic stability and was particularly associated with reduced postoperative arrhythmias and organ dysfunction. Whether the observed benefits derive from the monitoring technology, the structured hemodynamic algorithm, or their combination remains to be confirmed. Future multicenter studies should focus on refining hemodynamic targets for specific valve pathologies and should incorporate a protocolized conventional comparator arm to isolate the contributions of advanced monitoring and algorithmic care.

## Data Availability

The datasets of the current study are available from the corresponding author on reasonable request.
